# Application of Artificial Intelligence for Medical Research

**DOI:** 10.3390/biom11010090

**Published:** 2021-01-12

**Authors:** Ryuji Hamamoto

**Affiliations:** 1 Division of Molecular Modification and Cancer Biology, National Cancer Center Research Institute, 5-1-1 Tsukiji, Chuo-ku, Tokyo 104-0045, Japan; rhamamot@ncc.go.jp; 2 Cancer Translational Research Team, RIKEN Center for Advanced Intelligence Project, 1-4-1 Nihonbashi, Chuo-ku, Tokyo 103-0027, Japan

The Human Genome Project, completed in 2003 by an international consortium, is considered one of the most important achievements for mankind in the 21st century [1]. With the completion of this project, medical science has entered a new era known as the post-genome era [2]. In the latter half of the 20th century and into the 21st century, with the development of molecular biology, efforts to elucidate diseases at the molecular level were actively pursued worldwide. Under these circumstances, the completion of the whole human genome analysis has increased the momentum for personalized medicine, i.e., patient-specific medicine optimized by combining genome information and molecular medicine. Various technologies have been developed in the post-genome era; one of the major technological advances is the advent of next-generation sequencing (NGS). The duration of the human whole-genome analysis project undertaken by the international consortium was 13 years, costing USD 3 billion to analyze the entire genome of a single person [3]. However, with the advent of NGS, it now takes a day and less than USD 1000 for the same analysis [4]. Moreover, with the advent of such high-speed sequencers, the amount of data obtained in medical research is enormous, and the term “big data” is now common in medical research.

In the 21st century, with the progress of machine learning technology (especially the emergence of deep learning technology) and graphics processing units, big data analysis using artificial intelligence (AI) technology is now common in various fields, including the medical field [5]. Its introduction in the medical field allows for a more objective analysis of biological phenomena, which are inherently complex and diverse, and require careful determination of the generalizability of the results obtained from analyses. In such an academic field, if scientific discussions involve only limited data, then it becomes difficult to grasp the complete picture of a phenomenon, and it is easy to fall into a state of “you can’t see the forest for the trees.” In contrast, the analysis of large-scale data using AI technology will make it possible to elucidate biological phenomena more objectively and without omission; it is expected to contribute greatly to the advancement of medicine. In fact, more than 60 AI-powered medical devices are approved by the Food and Drug Administration (FDA) in the United States, and the use of AI in the medical field is trending worldwide [2].

As shown in Figure 1, there are three areas in which AI technology is currently implemented in the medical field: medical image analysis, omics analysis, and natural language processing [2,5,6,7,8,9]. In this Special Issue on the “Application of Artificial Intelligence for Medical Research”, articles were presented in the fields of medical image analysis and omics analysis, among these three areas. As the editor of this Special Issue, I would like to provide a brief overview of it.

Regarding radiation image analysis, Akatsuka et al. analyzed magnetic resonance images of the prostate with deep learning, compared them with observations by radiologists and pathologists, and showed that deep learning could identify cancerous areas at a high rate, and could also find useful clues for clinical diagnosis even when the cancer was not visible [10]. Sukegawa et al. confirmed that panoramic radiographs could be analyzed using a deep convolutional neural network to accurately classify dental implant systems [11]. Yamamoto et al. showed that hip radiographs could be analyzed using deep learning to diagnose osteoporosis with high accuracy, and that adding clinical covariates from patient records further improved performance [12]. For ultrasound image analysis, Dozen et al. proposed a new segmentation method called Cropping–Segmentation–Calibration, which is able to segment the ventricular septum in fetal ultrasound videos using time-series information [13]. Kusunose et al. analyzed echocardiography images with a convolutional neural network, and showed the possibility of classifying them into five standard views (long axis, short axis, two-chamber view, three-chamber view, and four-chamber view) [14]. As for skin image analysis, Jinnai et al. showed it is possible to accurately classify malignant tumors (malignant melanoma and basal cell carcinoma) and benign tumors (nevus, seborrhoeic keratosis, senile lentigo, and hematoma/hemangioma) by training a convolutional neural network on images of pigmented skin lesions [15].

In this Special Issue, new directions in medical image analysis using AI technology were also reported. Korb et al. measured serum samples using Fourier-Transform infrared spectroscopy and analyzed the results using deep learning to show its potential in discriminating between sera from healthy individuals, allergic patients, and patients treated with allergen-specific immunotherapy [16]. Aida et al. used conditional generative adversarial networks (CGANs) to segment cancer stem cells (CSCs) on phase-contrast images, and showed the potential for mapping CSC morphology to an undifferentiated state using a deep-learning CGAN workflow [17]. Kanada et al. proposed an efficient search method for identifying successful regions by exploring the parameters of coarse-grained molecular dynamic simulations using two machine learning methods: Bayesian optimization and active learning [18]. Yamato et al. proposed a segmentation method using deep learning to extract nerves from label-free endoscopic images obtained using coherent anti-Stokes Raman scattering for nerve-sparing surgery [19].

In terms of omics analysis, Tanaka et al. used Bayesian networks to construct an Epithelial–Mesenchymal Transition (EMT) network representing gene–gene interactions, and showed that the sample-specific edge contribution value pattern of this EMT network characterized the survival rate of lung cancer patients [20]. Using a publicly available dataset (The Cancer Genome Atlas (TCGA) with a focus on lung adenocarcinoma (LUAD)), Asada et al. succeeded in classifying good and poor prognosis groups by performing a multi-omics analysis combining deep learning and machine learning, and also successfully identified genes contributing to the survival of LUAD patients [21]. Takahashi et al. succeeded in classifying lung cancer patients based on their prognosis (poor or good) by analyzing a multi-omics data set consisting of six categories of TCGA using a combination of deep learning and machine learning [22]. Kobayashi et al. proposed a new method of adding per-element input scaling to diet networks, and showed that lung cancer pathological types (adenocarcinoma and squamous cell carcinoma) could be accurately identified using somatic mutation profiles [23]. Ai et al. analyzed microarray gene expression data using weighted gene co-expression network analysis and a variational autoencoder to predict colorectal cancer with high accuracy [24]. In their review, Lin et al. proposed the use of machine learning to improve the off-target properties of *N*-methylpyrrole-*N*-methylimidazole polyamides (pyrrole-imidazole polyamides (PIPs)) [25].

It is clear that AI is a useful technology in the medical field, and I believe it is imperative to keep abreast of the latest research findings to effectively utilize AI and overcome challenges in the future. I sincerely hope that this Special Issue will be of some support to readers.

## Figures and Tables

**Figure 1:**
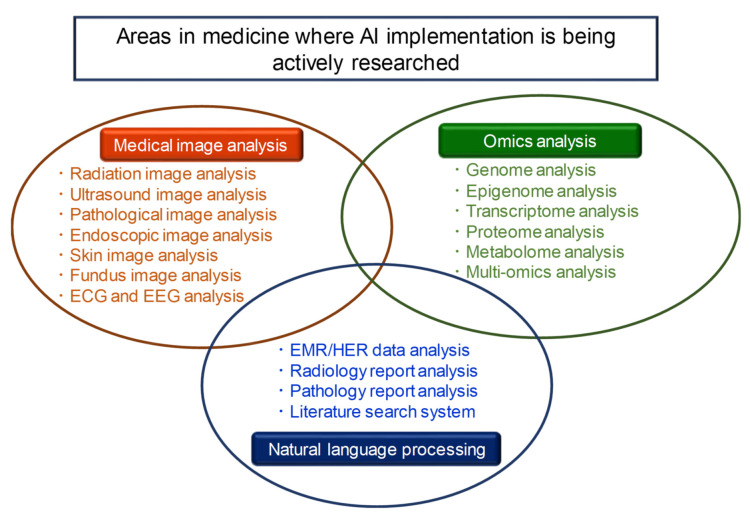
There are three areas in which AI technology is currently being actively implemented in the medical field: medical image analysis, omics analysis, and natural language processing.
